# What Drives Public Preference for Rare Drugs Coverage in China? Insights From a Multi‐Center Discrete Choice Experiment

**DOI:** 10.1111/hex.70735

**Published:** 2026-06-24

**Authors:** Ya'nan Wu, Jingdan Chen, Jiachen Shao, Hui Peng, Linkang Li, Gang Chen, Shunping Li

**Affiliations:** ^1^ Department of Social Medicine and Health Management, Cheeloo College of Medicine, School of Public Health Shandong University Jinan China; ^2^ NHC Key Lab of Health Economics and Policy Research (Shandong University) Jinan China; ^3^ Center for Health Management and Policy Research Shandong University (Shandong Provincial Key New Think Tank) Jinan China; ^4^ Center for Health Preference Research Shandong University Jinan China; ^5^ China Alliance for Rare Diseases Beijing China; ^6^ Melbourne School of Population and Global Health University of Melbourne Victoria Australia

**Keywords:** basic medical insurance, discrete choice experiment, orphan drugs, public preference, rare disease

## Abstract

**Introduction:**

Reimbursement decisions for orphan drugs are complex, as conventional cost‐effectiveness frameworks may not fully capture broader societal values. To investigate public preferences regarding the inclusion of rare disease drugs in China's Basic Medical Insurance (BMI) using a multi‐center discrete choice experiment.

**Methods:**

From July to September 2024, a face‐to‐face, filed‐based discrete choice experiment (DCE) was conducted across four regions in China. Using non probability sampling technique which is known as Quota sampling, participants evaluated drug profiles varying in six attributes. Mixed logit model and latent class model were used for analysis. Relative importance, willingness to pay (WTP), scenario analysis and preference heterogeneity were estimated.

**Results:**

Of the 761 respondents retained after initial data cleaning, 622 passed the internal consistency check and were included in the final analysis. Most participants were urban residents (62.7%), employed (66.6%), and covered by urban and rural resident basic medical insurance (BMI) (57.7%). The general public preferred including orphan drugs in BMI coverage when treatments provided greater health gains, targeted diseases with moderate untreated life expectancy, and lacked existing reimbursed alternatives. In contrast, poorer untreated quality of life and larger increases in BMI financing reduced utility. Health gains were the most important, followed by increases in BMI financing, untreated quality of life, existing BMI coverage, and untreated life expectancy. Annual WTP was ¥1.225 per capita for quality‐adjusted life years (QALY) gains (0.01–4), with lower WTP observed among respondents facing poorer untreated quality of life. Scenario analysis showed that 78.5% would support reimbursement for drugs providing maximal health gains (0.01–4). Preference heterogeneity was observed across age, sex, education, household income, insurance type, and urban–rural residence. Latent class analysis identified two subgroups. The “life‐saving group” (23%) showed strong preferences for greater disease severity and larger health gains, whereas the “pragmatist group” (77%) demonstrated relatively flatter preferences and negative utility associated with declining untreated life expectancy.

**Conclusion:**

While health gains drive preferences, the public also values equity, disease severity and affordability. Policymakers must integrate these multidimensional social values to ensure legitimacy and fairness in allocating finite resources for rare diseases.

**Patient or Public Contribution:**

Members of the public were involved in the development of the DCE. In the attribute development phase, members of the public participated in a pilot study to help ensure the clarity and comprehensibility of attribute descriptions and levels. And their feedback informed revisions to the questionnaire. In the main study, members of the public participated as respondents, providing data on societal preferences for orphan drug reimbursement.

**Abbreviations**
BMI: basic medical insurance
BWS: best‐worst scaling
CNY: Chinese Yuan
DCE: discrete choice experiment
LCA: latent class analysis
MIXL: mixed logit
NRDL: national reimbursement drug list
QALYs: quality‐adjusted life years
QoL: quality of life
RI: relative importance
SD: standard deviation
SE: standard error
UEBMI: urban Employee Basic Medical Insurance
UK: the United Kingdom
WTP: willingness to pay

## 1 Introduction

The pursuit of Universal Health Coverage requires policymakers to balance maximizing population health with ensuring equitable access for the most vulnerable groups [1]. Within this context, providing adequate care for rare diseases represents one of the most formidable challenges for health systems globally. Rare or Orphan diseases, which lack a universally accepted definition, are characterized by low prevalence rates, clinical heterogeneity, and potentially life‐threatening consequences [2]. There are 6000–8000 rare diseases globally, affecting over 400 million patients worldwide [3]. In China alone, there are over 20 million individuals living with a rare disease [4], many of whom face prolonged diagnostic delays, limited treatment options, and enormous financial burdens [5]. Rare/Orphan drugs are developed for these conditions [6]. An estimated 95% of rare diseases still lack approved therapies [7], and even available drugs are often inaccessible due to extreme prices [8] and restrictive reimbursement policies [9].

Ensuring access to orphan drugs is essential for health equity [10], with medical insurance being the primary mechanism [11]. However, the high cost of orphan drugs poses a moral and economic dilemma for public payers [12]. This tension reflects a fundamental conflict in drug pricing between utilitarian efficiency maximization and equity‐based ethical principles [13]. Notably, rarity alone is insufficient to justify premium pricing, and broader social value elements should be taken into account [14, 15, 16]. The equity‐efficiency tensions are widely shared across developed and developing countries facing rapid expansion of high‐cost technologies [17]. In China, while universal basic medical insurance (BMI) has expanded its National Reimbursement Drug List (NRDL) to include 95 orphan drugs by 2024 [11, 18], the current expert‐led selection process does not explicitly incorporate broader societal preferences, which may limit the perceived legitimacy and social acceptability of reimbursement decisions [19, 20]. As direct contributors to the BMI fund and representatives of healthy populations and patients with common diseases [21, 22], understanding the public's preferences [23] is essential for developing fair, effective, and socially acceptable policies [24].

Preferences can be elicited through qualitative, quantitative, or mixed‐methods approaches [25], with discrete choice experiments (DCEs) increasingly used in health economics [26] to capture stated preferences based on random utility theory [27]. To date, eight studies have used DCEs to examine preferences for orphan drug reimbursement among stakeholders, including the general public, patients with rare diseases, and policymakers [28, 29, 30, 31, 32, 33, 34, 35]. Among these, public preference is of the greatest concern, as they reflect societal values and contributes to the legitimacy of reimbursement decisions [28, 29, 34, 35]. However, several important gaps remain in the current literature. Existing evidence is predominantly derived from European settings, whose healthcare financing structures and social value frameworks differ substantially from those in China [36]. Moreover, most previous studies relied primarily on literature reviews and qualitative focus discussions for attribute development, with limited incorporation of public perspectives [30, 34]. Notably, the relative importance of attributes varies across studies, suggesting that value preferences for orphan drugs are context‐dependent and sensitive to study design. Evidence from China remains limited, and the only existing Chinese study was constrained by limited attribute development and convenience sampling [35], which may affect the robustness and contextual relevance of its findings.

Against this background, understanding public preferences is important for informing reimbursement decisions for orphan drugs within China's BMI scheme. Therefore, this study employed a DCE design with systematically developed attributes and levels to quantify public preferences, examine the trade‐offs individuals are willing to make across key reimbursement criteria, and explore preference heterogeneity across the population.

## 2 Materials and Methods

### 2.1 Sampling

A national multicenter cross‐sectional survey was conducted across urban and rural populations in four provinces (Shandong, Anhui, Guizhou, and Heilongjiang), to ensure broad geographic and socioeconomic representativeness. Quota sampling was used, stratifying respondents by age, gender, and urban‐rural residency. These quotas were based on China's Seventh National Population Census (2020) [37], enhancing the study's representativeness and generalizability.

The minimal sample size, derived using Johnson and Orme's method for DCE studies *N* > 500 × *c*/(*t* × *a*), where *c* represents the largest number of levels for any attribute, *t* represents the number of choice tasks, and a represents the number of alternatives per task [38]. Based on *c* = 5, *t* = 9, and *a* = 2, the minimum required sample size was 139. To improve geographic representativeness, quota sampling was implemented across provinces [37].

### 2.2 Discrete Choice Experiment

#### 2.2.1 Identification of Attributes and Levels

**Figure 1:**
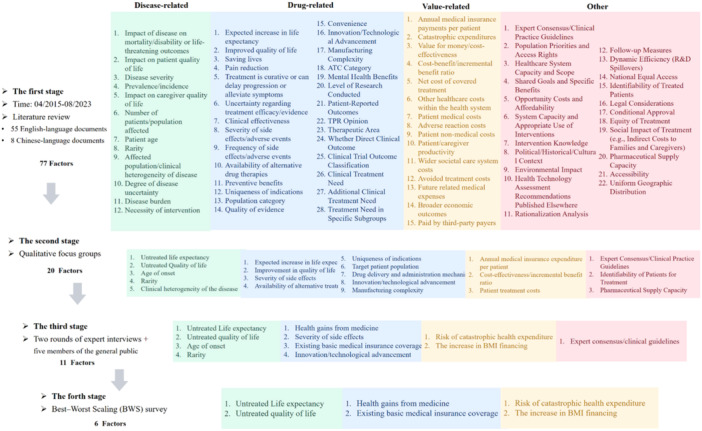
The four‐stage mixed‐methods attribute screening process.

The DCE design and analysis followed the International Society for Pharmacoeconomics and Outcomes Research good practice principles [39]. Attribute development is fundamental to DCEs. Traditionally, qualitative methods like literature reviews and expert consultations were used [40]. However, consensus on optimal methodology remains elusive [41]. Quantitative approaches, including Best‐Worst Scaling (BWS), are integrated for their high discriminative power [42]. Our study used a four‐stage mixed‐methods screening process (Figure 1), to identify key attributes and levels.

Initially, a thorough literature review was performed across 7 Chinese and English databases, employing terms including “rare disease,” “orphan drug,” “reimbursement,” and “health technology assessment.” The screening flowchart is in Supporting Information: Figure 1. Included studies frequently applied multi‐criteria decision analysis and DCE methods, reflecting perspectives from the public [30, 31, 35], patients [34] and decision‐makers [28]. Ultimately, 77 potential factors were extracted. The categorization of these factors was performed by three members of the research team through a process of discussion and consensus. Three research team members categorized these factors through discussion and consensus using an inductive approach, grouping conceptually similar items into four domains: disease‐related (12), drug‐related (28), value‐related (15), and other domains (22).

Secondly, qualitative focus group discussions with five experts (pharmacoeconomists, rare disease specialists, and research team members) reviewed and refined the attributes based on China's policy context, measurability, and DCE suitability, yielding 20 preliminary attributes. Thirdly, two rounds of expert interviews (with two health economics scholars, two medical insurance experts, and two rare disease specialists) were conducted to further refine the attribute list. And a pilot engagement with five members of the general public was undertaken to assess the face validity and readability. Based on this feedback, items were consolidated and modified. For instance, to improve comprehensibility, “cost‐effectiveness” was rephrased to “increase in BMI financing.” Ultimately, 11 attributes were examined: untreated life expectancy, untreated quality of life, age of onset, rarity, health gains, severity of side effects, existing BMI coverage, innovation/technological advancement, risk of catastrophic health expenditure, the increase in BMI financing, and expert consensus/clinical guidelines (Supporting Information: Table 1).

Finally, a BWS survey assessed the relative importance of the 11 attributes among 576 members of the public from 17 provinces in China, recruited through non‐probability quota sampling with age and gender quotas (Supporting Information: Table 2). In addition, the BWS respondents and the DCE respondents were independent samples and did not overlap. An object case (BWS‐1) approach [43] was implemented with a balanced incomplete block design [44]. Respondents completed 11 choice sets, each comprising 5 attributes, and selected the most and least important. Standardized investigator training and completion‐time screening were applied for quality control. Data were analyzed using counting methods and conditional Logit models. As shown in Supporting Information: Figure 2, based on standardized Best‐Worst scores and model‐derived relative importance, six attributes were ultimately identified. Importantly, these candidate attributes had already been identified as relevant and applicable to the Chinese reimbursement context through prior three stages. These were untreated quality of life, untreated life expectancy, health gains, the increase in BMI financing, risk of catastrophic health expenditure, and existing BMI coverage. Consistent with social preference theory, these reflect the public's concerns for both patient welfare and system sustainability in healthcare resource allocation.

The six attribute levels were systematically determined from multiple sources. Levels for health outcomes (“untreated quality of life,” “untreated life expectancy,” and “health gains”) were derived from empirical research [45, 46, 47], supplemented by clinical evidence [48, 49], and expert advice. The “increase in BMI financing” attribute was designed to capture willingness to pay, modeled as a per‐capita premium increase to reflect China's funding system. Culyer and Wagstaff [50] implied that equality in health can only be achieved through increased expenditure. This individual contribution was then translated into a national budget impact using official enrollment figures (approx. 1.35 billion [51]), a method supported by health insurance sustainability research [52, 53]. The United Kingdom (UK) studies on willingness to pay for rare drugs have used the additional income tax attribute [54]. Considering China's medical insurance funding system, it was more appropriate to reflect premium conditions using the per capita increase in medical insurance funding and the number of contributors. The levels of “the risk of catastrophic health expenditure,” and “existing BMI coverage” were determined based on the real‐world situations and expert consultations. Specific details are shown in Table 1.

**Table 1:** Attributes and associated levels identified for this discrete choice experiment.

Attribute (abbreviation)	definition	Level
Untreated quality of life (QOL)	The extent to which a rare disease affects the quality of life of a patient when the disease is present but untreated, 100% of the quality of life of a perfectly healthy person	High (90%) [Reference]
		Medium (60%)
		Low (30%)
		Very low (5%)
Untreated life expectancy (life)	Average length of time patients are expected to survive when they have a rare disease but are not receiving treatment	Mild (no effect on life expectancy) [Reference]
		Moderate (25 years of life expectancy after the disease)
		Severe (5 years of life expectancy after the disease)
		Very severe (0‐6 months to live after the disease)
Health gains (qaly)	The increase in quality‐adjusted life years (QALYs) that can be achieved by treating a disease with a particular drug, compared to standard of care or best supportive care	Average increase of 0.01 QALYs per patient [Reference]
		Average increase of 0.5 QALYs per patient
		Average increase of 1 QALY per patient
		Average increase of 2 QALYs per patient
		Average increase of 4 OALYs per patient
The increase in BMI financing (cost)	Average amount of additional financing per participant per year required for the inclusion of drugs for rare diseases outside the basic medical insurance	Average increase in annual Medicare financing of CNY 3 per person per year (approximately CNY 4.05 billion in additional Medicare funds per year) [Reference]
		Average annual Medicare financing increase of CNY 2 per person (approximately CNY 2.70 billion additional Medicare funds per year)
		Average annual increase of CNY 1 per person in health care financing (about CNY 1.35 billion increase in health care financing per year)
		Average annual increase of CNY 0.5 per person in health care financing (about CNY 680 million increase in health care financing per year)
		Average annual increase of CNY 0.2 per person in health care financing (about CNY 270 million increase in health care financing per year)
Risk of catastrophic health expenditure (huge)	Whether the medical expenses of patients with rare diseases result in catastrophic expenditures for the household, i.e., mandatory medical expenditures exceeding 40% of the household's general consumption	No [Reference]
		Yes
Existing BMI Coverage (drug)	Whether an existing treatment for the same rare disease was already covered by BMI	Approved drugs available [Reference]
		No approved drugs

#### 2.2.2 Experimental Design and Questionnaire Development

A ‐D‐efficient design was used to generate 18 choice tasks, which were then divided into 2 blocks (to be randomly presented to respondents) using Ngene software. A forced‐choice format (without an opt‐out or status quo option) was adopted to ensure that respondents made explicit trade‐offs between alternative reimbursement scenarios and to maximize the information available for estimating attribute preference weights. Supporting Information S1: Table 3 shows an example of a DCE task. In each DCE task, respondents were shown two hypothetical rare disease drugs and asked to choose, from a societal perspective, which one should be included in BMI given that only one could be reimbursed due to limited national medical insurance budgets. Additionally, within each block, a strictly dominant option, where option B was objectively superior to option A, was incorporated to assess the internal validity of responses.

The questionnaire comprised an introduction to the study context and definition of attributes, 11 DCE choice tasks, and sociodemographic questions. To help respondents be familiar with choice tasks, practice tasks were included before the formal choice tasks. A repeated choice task was inserted to ensure respondents' attention and consistent choices (i.e., consistency testing). Socio‐demographic information included gender, age, urban/rural, education level, marital status, work status, type of health insurance, annual household income, and fertility.

### 2.3 Data Collection

From July to September 2024, face‐to‐face data collection was conducted by trained interviewers. To ensure informed responses, respondents received a concise introduction to rare diseases, orphan drugs, and medical insurance, and were explicitly instructed to adopt a societal perspective. Detailed explanations of attributes and a practice task ensured full comprehension before the main questionnaire. Interviewers provided real‐time assistance, including reading questions aloud for those with visual or literacy challenges. Each 15–20 min survey concluded with a $6 compensation and direct return of questionnaires to ensure confidentiality.

To ensure response quality, exclusion criteria were defined as follows: (1) respondents whose completion time was below 300 s, (2) respondents who incorrectly answered dominant DCE questions, and (3) respondents who discontinued participation before completing the survey.

This study was approved by the Medical Ethics Committee of the Centre for Health Management and Policy Research at Shandong University (Ethics Approval No.: ECSHCMSDU20231001). And the research adhered to the tenets of the Declaration of Helsinki. All respondents provided their informed consent prior to their inclusion in the study.

### 2.4 Statistical Analysis

Data were analyzed using STATA (version 17.0). Descriptive statistics were used to summarize patient demographics, with categorical variables reported as frequencies and percentages, and continuous variables as means and standard deviations. For the DCE data, dummy coding was applied to assign attribute levels. The conditional logit model and mixed logit (MIXL) model were used to analyze the choice data.

The relative importance (RI) of each attribute was calculated using MIXL model estimates from the choice data set. The RI was assessed as the greatest difference between the coefficients associated with the levels of an attribute. A simulation approach was employed to estimate willingness to pay (WTP) by drawing 10,000 random samples from the distribution of the relevant parameters [55]. The ratios of parameter estimates were then calculated to derive individual WTP values. To improve robustness, extreme values in the WTP distribution (top and bottom 1%) were trimmed to mitigate their potential impact on the mean estimate. Scenario analyses were used to explore how respondents' probability of choosing a rare disease drug for inclusion in BMI varied with the level of one or more attributes.

Subgroup analyses were used to explore heterogeneity in public preferences and to test the applicability of social preference theory across different segments. The planned subgroups include geographical region, age, gender, education level, household income, urban vs rural residence, and type of medical insurance coverage. These subgroup divisions are commonly used in health preference and DCE research to explore potential variations in preference weights [56, 57]. By estimating conditional logit models within each subgroup, we can assess how attribute importance varies across social strata and examine whether preference manifest differently across population segments. To explore deeper preference differences beyond traditional subgroup divisions, this study further constructed a latent class analysis (LCA) model to identify latent respondent categories. All statistical analyses were conducted with a significance level of *α* = 0.05.

## 3 Results

### 3.1 Respondent Characteristics

A total of 810 individuals participated in the survey, of whom 805 completed the questionnaire. The mean (standard deviation, SD) completion time was 16.4 (1.68) minutes. After excluding 44 respondents, 1 residing outside the target provinces, 33 failing the dominance test, and 10 completing the survey in under 300 s, 761 respondents were retained for consistency assessment. Among them, 139 failed the internal consistency check and were excluded from the final analysis (Supporting Information S1: Table 4). Therefore, the final analytical sample consisted of 622 respondents. Table 2 summarizes the demographic characteristics of the analytical sample alongside benchmarks from the Seventh National Census (2020).

**Table 2:** Demographics of respondents included for analysis (*N* = 622).

Characteristic	All respondents No. (%)	Shandong province No. (%)	Guizhou province No. (%)	Heilongjiang province No. (%)	Anhui province No. (%)	Seventh population census of China, 2020 (%)
Sex						
Male	301 (48.4%)	143 (52.2%)	42 (42.4%)	64 (51.2%)	52 (41.9%)	51.2%
Female	321 (51.6%)	131 (47.8%)	57 (57.6%)	61 (48.8%)	72 (58.1%)	48.8%
Age, years						
20–29	93 (15.0%)	37 (13.5%)	16 (16.2%)	18 (14.4%)	22 (17.7%)	15.4%
30–39	120 (19.3%)	54 (19.7%)	16 (16.2%)	27 (21.6%)	23 (18.5%)	20.7%
40–49	120 (19.3%)	56 (20.4%)	22 (22.2%)	24 (19.2%)	18 (14.5%)	19.2%
50–59	128 (20.6%)	56 (20.4%)	20 (20.2%)	26 (20.8%)	26 (21.0%)	20.6%
60 and above	161 (25.9%)	71 (25.9%)	25 (25.3%)	30 (24.0%)	35 (28.2%)	24.1%
Geographical location						
Urban	390 (62.7%)	171 (62.4%)	66 (66.7%)	75 (60.0%)	78 (62.9%)	63.9%
Rural	232 (37.3%	103 (37.6%)	33 (33.3%)	50 (40.0%)	46 (37.1%)	36.1%
Education						
Elementary education	171 (27.5%)	54 (19.7%)	30 (30.3%)	48 (38.4%)	39 (31.5%)	NA
Secondary education	182 (29.3%)	85 (31.0%)	22 (22.2%)	41 (32.8%)	34 (27.4%)	NA
Lower stage of higher education (university < 4 years)	99 (15.9%)	51 (18.6%)	17 (17.2%)	9 (7.2%)	22 (17.7%)	NA
Higher education level (undergraduate and above)	170 (27.3%)	84 (30.7%)	30 (30.3%)	27 (21.6%)	29 (23.4%)	NA
Occupation						
Employed	414 (66.6%)	201 (73.4%)	63 (63.6%)	65 (52.0%)	85 (68.5%)	NA
Schoolchildren	53 (8.5%)	20 (7.3%)	6 (6.1%)	13 (10.4%)	14 (11.3%)	NA
Unemployed	106 (17.0%)	42 (15.3%)	22 (22.2%)	23 (18.4%)	19 (15.3%)	NA
Retired	49 (7.9%)	11 (4.0%)	8 (8.1%)	24 (19.2%)	6 (4.8%)	NA
Marital status						
Married	484 (77.8%)	230 (83.9%)	81 (81.8%)	84 (67.2%)	89 (71.8%)	NA
Unmarried	138 (22.2%)	44 (16.1%)	18 (18.2%)	41 (32.8%)	35 (28.2%)	NA
Having children, number						
0	123 (19.8)	43 (15.7%)	19 (19.2%)	27 (21.6%)	34 (27.4%)	NA
1	225 (36.2%)	111 (40.5%)	37 (37.4%)	49 (39.2%)	28 (22.6%)	NA
2 and more	274 (44.1%)	120 (43.8%)	43 (43.4%)	49 (39.2%)	62 (50.0%)	NA
Main types of health insurance						
Urban and rural residents basic medical insurance	342 (55.0%)	130 (47.4%)	56 (56.6%)	84 (67.2%)	72 (58.1%)	NA
Urban Employee Basic Medical Insurance	271 (43.6%)	142 (51.8%)	42 (42.4%)	37 (29.6%)	50 (40.3%)	NA
Commercial Medical Insurance	3 (0.5%)	0 (0.0%)	1 (1.0%)	2 (1.6%)	0 (0.0%)	NA
None	6 (1.0%)	2 (0.7%)	0 (0.0%)	2 (1.6%)	2 (1.6%)	NA
Annual household income, CNY 10,000						
< 3	152 (24.4%)	88 (32.1%)	8 (8.1%)	18 (14.4%)	38 (30.6%)	NA
3–8	191 (30.7%)	66 (24.1%)	28 (28.3%)	49 (39.2%)	48 (38.7%)	NA
8–15	168 (27.0%)	65 (23.7%)	35 (35.4%)	40 (32.0%)	28 (22.6%)	NA
15–25	77 (12.4%)	41 (15.0%)	22 (22.2%)	6 (4.8%)	8 (6.5%)	NA
25 and above	34 (5.5%)	14 (5.1%)	6 (6.1%)	12 (9.6%)	2 (1.6%)	NA

Abbreviation: NA, not available. NA indicates variables that were not used as quota sampling criteria.

The mean age was 47.3 ± 16.8 years. Over half of the participants (56.8%) had a primary or intermediate education. Urban residents accounted for 62.7% of the sample. Most respondents were married (77.8%) and employed (66.6%). Nearly all participants (98.6%) were covered by urban employee or urban–rural resident health insurance. More than half of the respondents (57.7%) reported an annual household income of Chinese Yuan (CNY) 30,000–150,000.

### 3.2 Model Estimates

No evidence of dominant preferences was observed. The mixed logit model showed better fit than the conditional logit model, with stable estimates achieved using 3500 random draws (Supporting Information S1: Figure 3).

As shown in Table 3, except for the attribute “the risk of catastrophic health expenditure”, the levels within each attribute were statistically different from the reference levels. Respondents preferred including orphan drugs in BMI coverage when the drugs provided substantial health gains (e.g., 4 quality‐adjusted life years [QALYs]), targeted rare diseases with moderate reductions in life expectancy (25 years), and lacked effective alternatives in the BMI catalog. Conversely, they exhibit aversion toward drugs used for lower untreated quality of life (e.g., reduced to 5% or 30%) and increase in BMI financing. Significant standard deviations for most attributes indicate notable preference heterogeneity. The alternative‐specific constant (asc1) was not statistically significant, suggesting no positional bias in choices.

**Table 3:** Mixed logit model estimates (*N* = 622).

	*β (SE)*	SD *(SE)*	WTP(CNY)/Summary WTP (million yuan)^a^
asc1	−0.056 (0.036)	0.003 (0.095)	
Untreated life expectancy (ref: Mild (no impact on life expectancy))			
Moderate (25 years of life expectancy after the disease)	0.180 (0.076)**	0.004 (0.227)	0.172 (232.20)
Severe (5 years of life expectancy after the disease)	−0.023 (0.072)	0.185 (0.255)	
Very severe (0–6 months to live after the disease)	0.030 (0.080)	1.099 (0.097)***	
Untreated quality of life (ref: 90%)			
Medium (60%)	−0.046 (0.075)	0.083 (0.388)	
Low (30%)	−0.257 (0.075)***	0.012 (0.168)	−0.245 (−330.75)
Very low (5%)	−0.330 (0.085)***	0.807 (0.096)***	−0.372 (−502.20)
Health gains (ref: 0.01QALY)			
0.5 QALY	0.190 (0.095)**	0.021 (0.248)	0.181 (244.35)
1 QALY	0.430 (0.087)***	0.009 (0.107)	0.409 (552.15)
2 QALY	0.728 (0.081)***	0.365 (0.151)**	0.687 (927.45)
4 QALY	1.297 (0.099)***	0.586 (0.132)***	1.225 (1653.75)
Risk of catastrophic health expenditure (ref: no)			
Yes	0.082 (0.049)*	0.732 (0.066)***	0.240 (324.00)
Existing BMI Coverage (ref: Approved drugs available)			
No approved drugs	0.285 (0.050)***	0.672 (0.068)***	
The increase in BMI financing	−0.188 (0.031)***	0.412 (0.046)***	

* *p* < 0.1

** *p* < 0.05

*** *p* < 0.01.

^a^ The attribute increase in BMI financing in this DCE was designed as the additional annual per‐capita contribution to basic medical insurance. Based on an estimated 1.35 billion contributors, these WTP estimates can be translated into the corresponding annual total increases in the insurance fund available for rare disease drug reimbursement.

### 3.3 Relative Attribute Importance

Health gains from medicine emerged as the most important attribute (49.5%) in the public preferences, followed by the increase in BMI financing (20.1%), untreated quality of life (12.6%), existing BMI coverage of other drugs (10.9%), and the untreated life expectancy (6.9%). The risk of catastrophic health expenditure attribute was of least importance and did not reach statistical significance. The relative importance of each attribute is shown in Supporting Information S1: Figure 4.

### 3.4 Willingness to Pay

As shown in Table 3, respondents placed the greatest value on health gains: moving from 0.01 to 4 QALYs corresponded to a WTP of 1.225 CNY per capita, equivalent to an annual increase of about 1.65 billion CNY in insurance funding (assuming 1.35 billion contributors). Untreated life expectancy also influenced preferences, with a shift from mild to moderate severity corresponding to a WTP of 0.172 CNY. By contrast, declines in untreated quality of life were associated with negative WTP values (–0.245 for a reduction from 90% to 30% and –0.372 for 90% to 5%), suggesting disutility. In addition, the absence of existing BMI coverage for a rare disease drug generated a positive WTP of 0.240 CNY, indicating that respondents favored expanding coverage to include treatments not yet reimbursed.

### 3.5 Scenario Analysis

Supporting Information S1: Table 5 presents the results of the scenario analysis. Compared with the baseline, when disease impact shifted from no reduction in life expectancy to a 25‐year reduction post‐onset, 54.5% (standard error [SE] = 0.019) of respondents expressed support. When untreated quality of life deteriorated below the reference level (30%/5%), the support rate declined (to 43.6%/41.8%). If the additional BMI financing requirement was lowered further to CNY 0.2 per person, the estimated support rose to 62.9%. Meanwhile, if the health gains of the drug increased from 0.01 QALYs to 4 QALYs, an estimated 78.5% (SE = 0.017) of respondents would support its inclusion in BMI. Whether or not the BMI already covered a medicine for the same rare disease also influenced support, 57.1% supported including the new drug in the BMI in the absence of coverage of an existing drug. And this support increased to 64.1% when the drug targeted conditions with moderate untreated life expectancy and no existing reimbursed alternatives in the BMI.

### 3.6 Preference Heterogeneity

Across all subgroups, health gains consistently carried the greatest weight in preferences, while other attributes varied notably. The details are shown in Supporting Information S1: Figure 5. Age stratification revealed younger individuals (20–29) prioritized life expectancy, those aged 30–49 favored existing BMI coverage, and respondents 50+ were most sensitive to increased BMI financing and untreated quality of life. Males placed more weight on untreated life expectancy than females. Lower‐educated and low‐family‐income groups showed heightened concern for increases in BMI financing, whereas higher‐income and higher‐educated groups ranked untreated quality of life and untreated life expectancy as their second priority. Within the Urban Employee Basic Medical Insurance (UEBMI) group, untreated quality of life was key, while untreated life expectancy carried no significant weight. Rural residents primarily prioritized the increase in BMI financing. In contrast, urban populations demonstrated a greater concern for fairness issues, specifically focusing on existing BMI coverage.

To uncover deeper preference heterogeneity hidden beneath these observable demographic characteristics, we further employed a Latent Class Analysis (LCA). Supporting Information S1: Figure 6 illustrates the two latent categories identified by the LCA model. Category 1 (“life‐saving group,” comprising 23%) exhibits exceptionally high preference sensitivity, prioritizing “untreated life expectance” and “health gains.” Utility values increase markedly with greater disease severity and potential benefits, reflecting a pronounced tendency to prioritize critical care. In contrast, category 2 “pragmatist group,” comprising 77%) exhibits overall flatter preferences. Its distinctive feature is a marked negative utility as untreated life expectancy decreasing.

## 4 Discussion

This is the first multicenter discrete choice experiment to investigate societal preferences for including orphan drugs in China's National BMI. In total, the Chinese public exhibits a profile best characterized as a “pragmatic rescuer.” The results reveal that the public places the greatest weight on health gains, followed by the increase in BMI financing, untreated quality of life, existing BMI coverage, and untreated life expectancy. In addition, our analysis identified different preference patterns among different subgroups. These findings carry important implications for policymakers in China and other countries striving to advance universal health coverage while confronting the growing budgetary pressures associated with high‐cost health technologies.

Our study's high qualification rate, likely due to face‐to‐face surveying [41], supports our forced‐choice design without an opt‐out alternative. Dhar and Simonson [58] caution that opting out may reflect cognitive effort reduction when no clearly superior option exists. Given the public's unfamiliarity with rare diseases, the complexity of task selection may elevate opt‐out selections [59]. This forced‐choice design is common in orphan drug coverage DCEs in Europe and China [28, 29, 35], and in most health economics DCEs [26]. While one UK‐based study examining DCE willingness to pay among ultra‐rare childhood disease did incorporate an opt‐out option, with researchers subsequently analyzing respondents' reasons for opting out to distinguish between genuine exit preferences and indifference responses when determining data inclusion in the analysis [54]. Overall, our forced‐choice approach ensures all respondents engage with the trade‐offs in orphan drug coverage decisions, providing clearer policy guidance.

Consistent with previous research findings [29, 35, 60], our cognitive interviews and BWS results showed that rarity is not a key influencing factor in the stage of determining attributes. Interestingly, according to the relative importance results, the most important attribute is health gains. This aligns with previous systematic review findings, which show that rarity is consistently rated as a low priority in public evaluations, while health benefits are valued more highly [61]. Ethically, this aligns with a broader view in health economics and bioethics that utilitarian benefit maximization and egalitarian fairness, rather than disease prevalence, should inform public funding decisions [62, 63].

The increase in BMI financing attribute was also important. Unlike previous studies that primarily focused on the total annual costs [28, 30, 35], this study adopted a more comprehensive approach by using the per capita increase in basic health insurance financing. This methodological shift offers a more intuitive and comparable measure that directly reflects the additional financial burden borne by individual enrollees, which may be more relevant to both policymakers and the public.

Notably, the risk of catastrophic health expenditure was not a significant factor for the general population. Evidence from a study of seven rare diseases in China shows that the proportions of patients experiencing catastrophic health expenditure are under 0.167‰ [64]. In this study, 98.3% of respondents were covered by BMI, and those without rare diseases experience might have a particularly low perception of potential catastrophic costs. Empirical research suggests that insured individuals exhibit higher risk tolerance in financial decisions, reflecting reduced anxiety about large medical expenses [65]. Notably, individuals with university education and middle family income viewed catastrophic medical expenditures as statistically significant, unlike the full sample. This finding suggests that the primary barrier to promoting inclusive or mutual‐aid–based rare disease protection mechanisms in China lies in limited public awareness and understanding, rather than financial affordability alone. As this study focuses on the general public, future research could extend preference measurement to other groups to explore the weight of this attribute among those with real‐world experience.

The observed preference patterns for the disease‐related attributes, untreated life expectancy and untreated quality of life, highlight the public's non‐linear preferences and threshold effects. Similar patterns have been documented in prior Chinese studies [35]. Consistent with health resource allocation research, respondents did not show any particular preference for patients at end‐of‐life or with the poorest health status [66]. Our study found aversion toward a very poor quality of life (QoL) (30% and 5%) but no statistical significance for untreated life expectancy levels of 5 years or ≤ 6 months. This indicates a substantially stronger aversion to severe deterioration in quality of life than to reduced life quantity. Similar findings appear in societal QALY preferences research. For example, Lancsar et al. reported that when QoL is very low (e.g., 5%), improvements in QoL are preferred over equivalent life‐extension QALYs [45]. These findings imply that in rare disease policy, orphan drug coverage may best align with public values when targeting conditions involving substantial life expectancy extension or avoiding extremely poor quality of life.

Our findings indicate substantial variation in WTP for rare disease drug coverage across attributes, reflecting both efficiency and equity considerations. Respondents showed higher WTP for drugs treating diseases with moderate reductions in untreated life expectancy (25 years), but WTP fell sharply when the untreated quality of life was severely impaired (30% or 5%). This finding enriches social preference theory and the diminishing “rule of rescue” effect [67], suggesting altruism toward vulnerable groups is constrained by perceived outcome value. However, a review on attributes in health care priority settings suggests greater preference for the more severely ill [68]. WTP was positively associated with QALY gains, aligning with international findings that incremental improvements strongly influence societal valuation [30, 69]. Moreover, respondents favored coverage for drugs without prior BMI reimbursement, reflecting concerns for fairness and access [70]. Scenario analyses further showed that public support increased as required BMI financing decreased, underscoring the importance of affordability in insurance expansion [71].

Our findings revealed considerable preference heterogeneity across demographic subgroups, consistent with a systematic review on factors influencing WTP for health insurance in low‐ and middle‐income countries [72]. From a social preference theory perspective, subgroup differences highlight how values such as patient welfare (untreated quality of life, life expectancy), equitable access (existing BMI coverage), efficiency (health gains), and system sustainability (increase in BMI financing) shape judgments about coverage decisions. This profound conflict in value orientations, as evidenced by the identification of two distinct latent groups, underscores challenges facing health resource allocation. Successful policies must strike a delicate balance between efficiency and equity, evidence‐based decision‐making and compassion‐driven interventions. While responding to the pragmatic tendencies of the majority, mechanisms such as dedicated funds or specific evaluation pathways could be established to address the socio‐moral demand for “saving lives” (the rule of rescue) [73].

Several limitations should be acknowledged in our study. First, although quota sampling based on age, sex, and urban–rural distribution was applied, the use of non‐probability sampling may limit the national representativeness of the findings. Second, as with all stated‐preference studies, respondents' choices in hypothetical scenarios may not fully reflect real‐world reimbursement preferences, introducing potential hypothetical bias. Third, we did not employ visual methods to illustrate attribute levels, although we provided qualitative and quantitative descriptions of each level and real‐time interviewer support. Fourth, face‐to‐face interviews enhanced understanding and data quality, but the structured format constrained attribute order randomization. Future research should explore computer‐assisted or web‐based methods to balance guided collection with randomization. Fifth, the forced‐choice DCE design did not include an opt‐out or status quo alternative. Although this approach facilitated the elicitation of trade‐offs across reimbursement attributes, it may not fully capture real‐world reimbursement decision‐making contexts and could potentially overestimate support for orphan drug inclusion in BMI, particularly in scenario analyses and WTP estimates. Finally, the completion of 11 DCE choice tasks may have imposed a substantial cognitive burden on some respondents, potentially leading to response fatigue and less consistent preference elicitation. Although an internal consistency check was implemented to enhance data quality, some residual inconsistency in responses may still have remained.

## 5 Conclusion

Our study, using a discrete choice experiment, reveals that public preferences for orphan drug reimbursement in BMI are driven by multifaceted social values. Health gains are the primary driver. Additionally, increased BMI financing, untreated quality of life, existing BMI coverage, and untreated life expectancy significantly influence preferences. Heterogeneity in social preferences was observed, emphasizing the need for context‐specific reimbursement strategies. The findings highlight the need for incorporating societal values and preference heterogeneity into more flexible and equitable reimbursement policies.

## Author Contributions

**Ya'nan Wu:** methodology, formal analysis, data curation, writing – original draft, visualization. **Jingdan Chen:** methodology, writing – original draft. **Jiachen Shao:** investigation. **Hui Peng:** investigation. **Linkang Li:** writing – review and editing. **Gang Chen:** writing – review and editing, supervision. **Shunping Li:** conceptualization, project administration, supervision, funding acquisition. All authors certify that they meet the ICMJE criteria for authorship.

## Conflicts of Interest

Ya'nan Wu, Jingdan Chen, Jiachen Shao, Hui Peng, Linkang Li, Gang Chen, and Shunping Li have no conflicts of interest that are directly relevant to the content of this article.

## Supporting information

Supporting File

## Data Availability

The data are not publicly available due to privacy or ethical restrictions. The data supporting the findings of this study are available from the corresponding author upon reasonable request.
