# Supplementary material for: What Drives Public Preference for Rare Drugs Coverage in China? Insights From a Multi‐Center Discrete Choice Experiment
Source: Health Expect. 2026 Jun 24;29(4):e70735. doi: 10.1111/hex.70735 (PMC13292014; doi:10.1111/hex.70735)
Supplement: Supplementary file 1 — Supporting File [file HEX-29-e70735-s001.docx]

**eTable 1 Attributes and definitions included in the BWS study**

| attribute | definition |
| --- | --- |
| Untreated life expectancy | Average length of time patients are expected to survive when they have a rare disease but are not receiving treatment |
| Untreated quality of life | The extent to which a rare disease affects the quality of life of a patient when the disease is present but untreated, 100% of the quality of life of a perfectly healthy person |
| Age of onset | The average age at which the disease occurs in patients. |
| Rarity | The average prevalence of the disease. |
| Health gains | the increase in quality-adjusted life years (QALYs) that can be achieved by treating a disease with a particular drug, compared to standard of care or best supportive care |
| Severity of side effects | The severity of adverse effects that may occur following administration of a particular medication |
| Existing basic medical insurance coverage | Whether an existing treatment for the same rare disease was already covered by BMI |
| Innovation/technological advancement | The innovativeness of a drug, such as whether it fills a gap in national clinical treatment, addresses unmet clinical needs, or drives domestic independent research and development, and other assessments of its innovative value. |
| Risk of catastrophic health expenditure | Whether the medical expenses of patients with rare diseases result in catastrophic expenditures for the household, i.e., mandatory medical expenditures exceeding 40% of the household's general consumption |
| The increase in BMI financing | Average amount of additional financing per participant per year required for the inclusion of drugs for rare diseases outside the basic medical insurance |
| Expert consensus/clinical guidelines | Whether the drug is supported by expert consensus statements or recommended in clinical practice guidelines for its target disease. |

**eTable2 Demographic characteristics of respondents included in the Best-Worst Scaling analysis (N=576)**

| Variable | Number | Proportion (%) |
| --- | --- | --- |
| **Age (years)** | | |
| Minimum–Maximum | 18-84 |  |
| Mean (Standard Deviation) | 46.4（±15.8） |  |
| **Gender** | | |
| male | 292 | 50.7 |
| female | 284 | 49.3 |
| **location** | | |
| urban | 468 | 81.3 |
| rural | 108 | 18.8 |
| **Marriage status** | | |
| Married | 437 | 75.9 |
| Unmarried | 117 | 20.3 |
| Divorced | 7 | 1.2 |
| Widowed | 15 | 2.6 |
| **Educational level** |  |  |
| Primary school and below | 50 | 8.7 |
| Lower secondary school or vocational secondary school | 133 | 23.1 |
| Upper secondary school | 68 | 11.8 |
| Higher national diploma | 81 | 14.1 |
| Undergraduate degree and above | 157 | 27.3 |
| Postgraduate degree and above | 87 | 15.1 |
| **Occupation** | | |
| In employment | 449 | 78.0 |
| Unemployed | 32 | 5.6 |
| Retired | 95 | 16.5 |
| **Household income (**CNY 10,000) | | |
| ＜3 | 57 | 9.9 |
| 3–8 | 135 | 23.4 |
| 8–15 | 150 | 26.0 |
| 15–25 | 112 | 19.4 |
| ≥25 | 122 | 21.2 |
| **Main types of health insurance** | | |
| Urban and rural residents basic medical insurance | 256 | 44.4 |
| Urban Employee Basic Medical Insurance | 289 | 50.2 |
| others^a^ | 31 | 5.4 |

**eTable 3 the example of a DCE task**

|  | **Rare Drug 1** | **Rare Drug 2** |
| --- | --- | --- |
| untreated life expectancy | Mild (no impact on life expectancy) | Moderate (25 years of life expectancy after the disease) |
| untreated quality of life | Medium (60%) | Very low (5%) |
| health gains | Average increase of 4 OALYs per patient | Average increase of 0.5 OALYs per patient |
| the increase in BMI financing | Average annual increase of ￥0.2 per person in health care financing  (about ￥4.05 billion increase in health care financing per year) | Average annual Medicare financing increase of ￥2 per person (approximately ￥680 million additional Medicare funds per year) |
| Risk of catastrophic health expenditure | No | Yes |
| Existing BMI Coverage | Approved drugs available | No approved drugs |
| Which drug do you believe should be prioritized for inclusion in basic health insurance coverage? | ○ | ○ |

**eTable 4 The sensitivity analysis based on conditional logit model**

|  | **ALL sample (N=761)** | |  | **Exclude samples that failed the consistency test (N=622)** | |
| --- | --- | --- | --- | --- | --- |
|  | ***β（SE）*** | ***P*** |  | ***β（SE）*** | ***P*** |
| asc1 | -0.029 (0.020) | 0.149 |  | -0.027 (0.023) | 0.229 |
| Untreated life expectancy (ref: Mild) |  |  |  |  |  |
| Moderate (25 years of life expectancy after the disease) | 0.192 (0.047) | <0.001 |  | 0.142 (0.052) | 0.007 |
| Severe (5 years of life expectancy after the disease) | 0.077 (0.052) | 0.134 |  | 0.038 (0.057) | 0.502 |
| Very severe (0-6 months to live after the disease) | 0.071 (0.055) | 0.199 |  | 0.042 (0.061) | 0.487 |
| Untreated quality of life (ref: 90%) |  |  |  |  |  |
| Medium (60%) | -0.018 (0.041) | 0.672 |  | 0.000 (0.047) | 0.999 |
| Low (30%) | -0.112 (0.048) | 0.019 |  | -0.130 (0.053) | 0.014 |
| Very low (5%) | -0.132 (0.056) | 0.019 |  | -0.168 (0.063) | 0.007 |
| Health gains (ref: 0.01QALY) |  |  |  |  |  |
| 0.5 QALY | 0.182 (0.056) | 0.001 |  | 0.085 (0.062) | 0.168 |
| 1 QALY | 0.277 (0.048) | <0.001 |  | 0.254 (0.052) | <0.001 |
| 2 QALY | 0.526 (0.049) | <0.001 |  | 0.489 (0.052) | <0.001 |
| 4 QALY | 0.845 (0.055) | <0.001 |  | 0.849 (0.060) | <0.001 |
| Risk of catastrophic health expenditure (ref: no) |  |  |  |  |  |
| Yes | 0.070 (0.032) | 0.029 |  | 0.060 (0.035) | 0.090 |
| Existing BMI Coverage (ref: Approved drugs available) |  |  |  |  |  |
| No approved drugs | 0.191 (0.030) | <0.001 |  | 0.203 (0.033) | <0.001 |
| The increase in BMI financing | -0.108 (0.017) | <0.001 |  | -0.124 (0.020) | <0.001 |
| Observation | 13698 | |  | 11196 | |
| Loglikelihood | -4507.2998 | |  | -3672.36 | |
| AIC | 9042.6 | |  | 7372.72 | |
| BIC | 9147.95 | |  | 7475.246 | |

**eTable 5 Estimated uptake of hypothetical incentive profiles compared with base profile**

|  | **Base**  **profile** | **profile 1** | **profile 2** | **profile 3** | **profile 4** | **profile 6** | **profile 7** | **profile 8** | **profile 9** | **profile 10** |
| --- | --- | --- | --- | --- | --- | --- | --- | --- | --- | --- |
| Untreated life expectancy | mild | **moderate** | mild | mild | mild | mild | mild | mild | **moderate** | **moderate** |
| Untreated quality of life | 90% | 90% | **30%** | **5%** | 90% | 90% | 90% | 90% | 90% | **30%** |
| Health gains | 0.01 | 0.01 | 0.01 | 0.01 | **1** | **4** | 0.01 | 0.01 | 0.01 | **2** |
| The increase in BMI financing | 3 | 3 | 3 | 3 | 3 | 3 | **0.2** | 3 | 3 | **1** |
| Existing BMI coverage | Yes | Yes | Yes | Yes | Yes | Yes | Yes | **No** | **No** | **No** |
| Estimated uptake of hypothetical profile, No. (SE) | NA | 0.545  (0.019) | 0.436  (0.018) | 0.418  (0.021) | 0.606  (0.021) | 0.785  (0.017) | 0.629  (0.020) | 0.571  (0.012) | 0.641  (0.020) | 0.743  (0.023) |

Relevant literature retrieved through database searches (n=8473):

PubMed (n=1452), Web of Science (n=1702), Scopus (n=3329), CNKI (n=495), VIP Database (n=569), Wanfang Data Platform (n=496), CBM Database (n=430)

Obtained relevant literature through other supplementary resources (n=0)

After removing duplicates, the number of documents obtained was (n=4497)

Initial screening (n=4497)

Rescreening (n=3877)

Excluded (n=620)

Non-Chinese/English (n=20)

Literature without authors or

abstracts (n=600)

Literature included in the analysis (n=63)

Chinese literature (n=8)

English literature (n=55)

Excluded (n=3814)

Literature unrelated to the topic (n=2595)

Clinical literature not referencing HTA

(n=991)

General literature describing the orphan

drug environment without referencing HTA

(n=226)

Original text unavailable (n=2)

**eFigure 1 The literature screening flowchart**

Note: Searches for Chinese literature were conducted in databases including China National Knowledge Infrastructure (CNKI), Wanfang Data Platform, VIP Database, and CBM Chinese Database. Keywords primarily included: “rare disease,” “orphan drug,” “medical insurance,” “medical insurance reimbursement,” “evaluation,” “health technology assessment,” “resource allocation efficiency,” “threshold,” “decision-making,” “policy,” and “evidence.” The search period was restricted from the establishment of each database to August 31, 2023. English-language literature was retrieved from PubMed, Web of Science, and Scopus databases. Building upon the research of Tamás Zelei et al.^[[1]](#footnote-0)^. Search keywords were defined as: “rare disease,” “orphan,” “reimburse,” “evaluation,” “effective,” “access,” “HTA,” “threshold,” “decision,” “policy,” and “evidence”. The search period was set from April 1, 2015, to August 31, 2023. Specific search strategies varied across databases.

**eFigure 2 The Best-worst scaling results**

**eFigure 3 Estimation Iterations of the Mixed Logit Model**

Note: MNL, multinomial logit model; MIXL, mixed logit model.

**
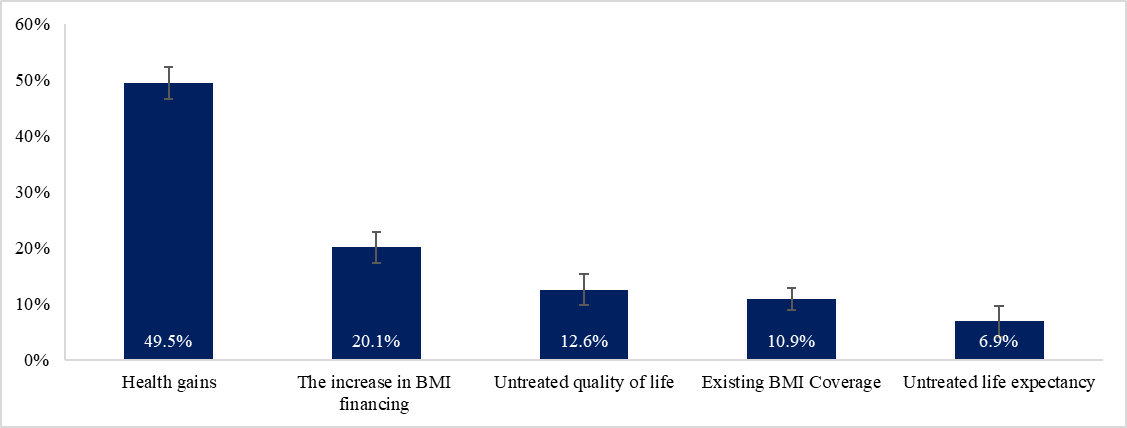
**

**eFigure 4 Attribute relative importance in full-sample measurement based on conditional logit model**

| 1. age group |
| --- |
| 1. gender |
| 1. educational level |
| 1. household income |
| 1. Basic medical insurance |
| 1. urban vs rural residence |

**eFigure 5 Attribute Relative Importance Through Subgroup Analysis based on conditional logit models**

Note: UEBMI: Urban Employee Basic Medical Insurance; URRMI: Urban and Rural Residents' Medical Insurance


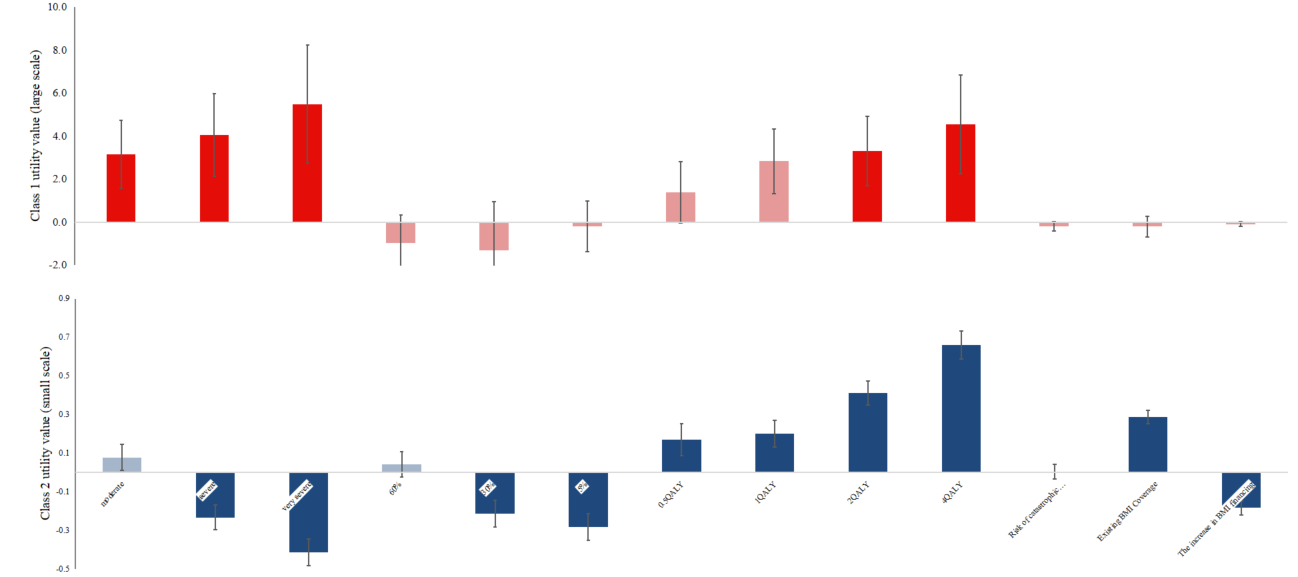


**eFigure 6 The latent class model results.**

Note: Dark-colored bars indicate coefficients that are statistically significant (*P* < 0.05). Error bars represent standard errors.

1. Zelei T, Molnár MJ, Szegedi M, Kaló Z. Systematic review on the evaluation criteria of orphan medicines in Central and Eastern European countries. Orphanet J Rare Dis. 2016 Jun 4;11(1):72. doi: 10.1186/s13023-016-0455-6 [↑](#footnote-ref-0)
